# A New Framework for Interpreting the Outcomes of Imperfectly Blinded Controlled Clinical Trials

**DOI:** 10.1371/journal.pone.0048984

**Published:** 2012-12-06

**Authors:** Ognjen Arandjelović

**Affiliations:** Centre for Pattern Recognition and Data Analytics, School of Information Technology, Deakin University, Geelong, Australia; University of Michigan, United States of America

## Abstract

It is well known that the outcome of an intervention is affected both by the inherent effects of the intervention and the patient's expectations. For this reason in comparative clinical trials an effort is made to conceal the nature of the administered intervention from the participants in the trial i.e. to *blind* the trial. Yet, in practice perfect blinding is impossible to ensure or even verify *post hoc*. The current clinical standard is to follow up the trial with an auxiliary questionnaire, which allows trial participants to express in closed form their belief concerning the intervention, i.e. trial group assignment (treatment or control). Auxiliary questionnaire responses are then used to compute the extent of blinding in the trial in the form of a *blinding index*. If the estimated extent of blinding exceeds a particular threshold the trial is deemed sufficiently blinded; otherwise, the strength of evidence of the trial is brought into question. This may necessitate that the trial is repeated. In this paper we make several contributions. Firstly, we identify a series of problems of the aforesaid clinical practice and discuss them in context of the most commonly used blinding indexes. Secondly, we formulate a novel approach for handling imperfectly blinded trials. We adopt a feedback questionnaire of the same form as that which is currently in use, but interpret the collected data using a novel statistical method, significantly different from that proposed in the previous work. Unlike the previously proposed approaches, our method is void of any *ad hoc* free parameters and robust to small changes in the participants' feedback responses. Our method also does not discard any data and is not predicated on any strong assumptions used to interpret participants' feedback. The key idea behind the present method is that it is meaningful to compare only the corresponding treatment and control participant sub-groups, that is, sub-groups matched by their auxiliary responses. A series of experiments on simulated trials is used to demonstrate the effectiveness of the proposed approach and its superiority over those currently in use.

## Introduction

Ultimately, the main aim in a clinical trial is straightforward: it is to examine and quantify the effectiveness of a treatment of interest. Effectiveness is evaluated relative to the effectiveness of a particular reference, the so-called *control* intervention. The form that the control intervention takes is dependent on the nature of the treatment which is studied. Since the goal of any newly proposed treatment is to better those which are already available and practiced, the common standard is to make the comparison with the current best alternative. In some instances this may be no active intervention at all.

### Controlling and Blinding Trials

To ensure that the aforementioned comparison is meaningful, it is of essential importance to ensure that any factors not inherently associated with the two interventions (treatment a`nd control) are normalized (controlled) between the two groups. This ensures that the observed differential outcome truly is the effect of differing interventions rather than any orthogonal, confounding variables.

A related challenge is that of *blinding* (or masking). Blinding refers to the concealment of the type of administered intervention from the individuals/patients participating in a trial and its aim is to eliminate differential placebo effect between groups [1]–[3]. Although conceptually simple, the problem of blinding poses difficult challenges in a practical clinical setup. We highlight two specific challenges which most strongly motivate the work of the present paper. The first of these stems from the difficulty of ensuring that absolute blinding with respect to a particular trial variable is achieved. The second challenge arises as a consequence of the fact that blinding can only be attempted with respect to those variables of the trial which have been identified as revealing of the treatment administered. Put differently, it is always possible that a particular variable which can reveal the nature of the treatment to a trial participant is not identified by the trial designers and thus that no blinding with respect to it is attempted or achieved. This is a ubiquitous problem, present in every controlled trial, and one which can severely affect the trial's outcome.

### Assessing Blinding

Given that it is both practically and in principle impossible to ensure perfect blinding, the practice of assessing the level of blinding after the commencement of a trial has been gaining popularity and general acceptance by the clinical community [4], [5]. The key idea is to use a statistical model and the participants' responses to a generic questionnaire to quantify the participants' knowledge about the administered intervention. While the statistical model used to this end has been a source of disagreement between researchers, as discussed in detail in the “Previous Work” section, the general approach is shared by different methods described in the literature. In this paper we argue that this common approach suffers from several important limitations:

- it necessitates the inclusion of *ad hoc* free parameters in the underlying statistical model,
- the assumptions underlying the interpretation of the auxiliary questionnaire responses are not universally upheld,
- the blinding assessment can be highly sensitive to small changes in the participants' questionnaire responses, and
- it leads to a sequential separation of inference concerning the extent of trial blindness and the assessment of differential effectiveness of the treatment.

Motivated by these key limitations of previous work, in the present work we propose a novel statistical framework and use it to derive an original method for integrated trial assessment which is experimentally shown to produce more meaningful and more clearly interpretable data. One of the key ideas behind the present method is that it is meaningful to compare only the corresponding treatment and control participant sub-groups, that is, sub-groups matched by their auxiliary responses. The inference of the differential effect of treatment is then achieved through Bayesian analysis.

### Paper Organization

The remainder of this paper is organized as follows. In the next section we describe the design of auxiliary data collection and review the most influential methods in the literature which use this data to assess participants' blindness in a trial. The main limitations of previous work are discussed in detail in this section as well. The proposed methodology, the conceptual idea behind it and the key mathematical formulae, are introduced subsequently. This is followed by a series of experiments which are used to illustrate systematically the advantages of our method. Finally, a further discussion of experimental results and the accepted clinical practices is presented before the manuscript is concluded with a summary of our main contributions and the possible avenues for further work.

## Previous Work

In this section we describe the general methodology of auxiliary data collection, the two most influential statistical models which use the aforesaid data to quantify the extent of blinding in a trial, and discuss the key limitations of the existing approaches which motivate the work described in the present paper.

### Method 1: James's Blinding Index

At the heart of the so-called *blinding index* proposed by James *et al.* [6] is the observation that the effect of a particular intervention is affected by the participant's perception of the effectiveness of the intervention the participant believes was administered. For example, a control group member who incorrectly believes to be a member of the treatment group may indeed experience positive effects expected from the studied treatment. The is the well-known and extensively studied placebo effect [7], [8]. Similarly, a treatment group member who incorrectly believes to be a member of the control group may experience treatment effects less pronounced than in the case of the correct assignment guess, or indeed than in the case of absence of a belief either way.

#### Design of Auxiliary Data Collection

James *et al.* propose the use of a post-trial questionnaire (the contentious issue of the timing of the questionnaire is discussed in the “Interpretation of Participants' Feedback” section) to assess the level of blinding in the trial. Following the trial, in its basic form the questionnaire asks the trial participants to state if they believe that they were assigned to:

- the control group,
- the treatment group, or
- if they are uncertain of their membership (the “don't know” response).

Extensions of this scheme which attempt to harness more detailed information have also been used, for example allowing the participants to quantify the conviction of their belief as “weak” or “strong”. In that case, the questionnaire would offer five choices:

- strong conviction of belonging to the control group,
- weak conviction of belonging to the control group,
- strong conviction of belonging to the treatment group,
- weak conviction of belonging to the treatment group,
- uncertain membership (the “don't know” response).

More granular auxiliary data choices have the potential of providing a more accurate picture of the extent of blinding. However, depending on the statistical model used, this advantage may come at the cost of reduced statistical significance for each of the response sub-groups (see the “Methods” section).

#### Quantifying the Extent of Blinding

For the sake of clarity of presentation, we use the same mathematical notation throughout the paper. The key symbols and their meanings are summarized for the benefit of the reader in Table 1.

**Table 1:** Notational convention for mathematical symbols adopted in this paper.

Symbol	Description
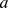	subscript modifier specifying group assignment; 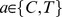 : 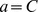 signifies control group assignment and 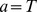 signifies treatment group assignment
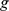	subscript modifier specifying membership belief; 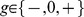 : 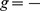 signifies belief in control group membership, 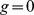 signifies uncertainty and 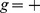 signifies belief in treatment group membership
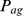	proportion of participants who were assigned to the group 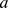 and who believe their membership to be 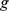
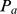	proportion of participants who were assigned assigned to the group 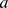
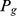	proportion of participants who believe their group membership to be 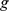
height 4	

The existing work on the assessment of blinding in trials uses the collected auxiliary responses to calculate a statistic referred to as the blinding index. For a 3-tier auxiliary questionnaire, James *et al.* [6] define their blinding index 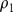 as:
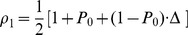
(1)It can attain values in the interval 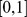, higher values denoting increasing levels of blindness. Thus 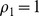 indicates perfect blinding and 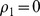 an unblinded trial. It can be seen that 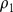 in Equation 1 comprises two non-constant terms. The first of these is the estimate 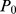 of the probability of the “don't know” response. From the collected questionnaire responses, 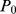 can be computed as follows:
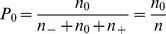
(2)where 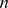 is the total number of participants in the trial, and 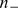, 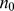 and 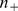 respectively the numbers of participants who believe that they were assigned to the control group, those who were uncertain of their assignment and those who believe that they were assigned to the treatment group. As the value of 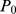 is increased so is its contribution to the blinding index through the first term in Equation (1). This fits the intuition that in a perfectly blinded trial participants should be entirely ignorant of the group they were assigned to, that is to say, of the intervention they were administered.

**Figure 1:**
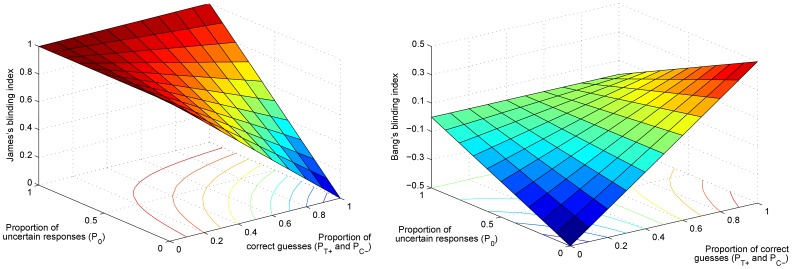
Plot showing the dependency of the blinding indexes (a) 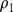 proposed by James *et al.* [6] and (b) 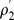 proposed by Bang *et al.* **** [9] on the proportions of “don't know” responses 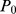, and the correct assignment guesses 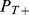 and 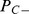. Note that although 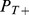 and 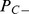 are independent variables, due to their symmetric contributions and for the purpose of easier visualization, in this plot it was taken that 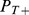 and 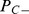 were always equal (making the plot readily representable in 3D instead of 4D).

The second term contributing to the blinding index is proportional to the statistic 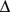 which takes into account the distribution of participants who do have a positive or negative belief regarding their assignment, that is, who believe to belong to either the control or the treatment group. James *et al.* define 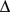 as:
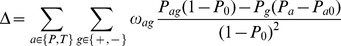
(3)In Equation (3), the constants 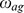 are weighting coefficients. In this expression their effect is to scale relative contributions of the correct and incorrect group assignment guesses. To gain intuitive insight into the nature of the 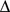 statistic, consider the plot shown in Figure 1(a). From the plot, it is readily apparent that 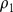 is a concave function which attains its maximal value of 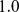 when (i) all participants are uncertain of their assignment (i.e. 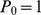) or (ii) when all participants have an incorrect belief regarding their assignment (i.e. 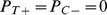). Expressed formally:
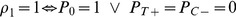
(4)In comparison with the case of 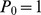 the attainment of the maximal value 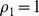 for 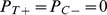 is more questionable. While it is tempting to reason that blinding must have been successful since no participant correctly guessed their assignment, it would be erroneous to do so. In particular, the *consistency* of the wrong belief amongst trial participants actually reveals unblinding, but with the participants' incorrect association of the unblinded factor with the corresponding group assignment. For example, the treatment may cause perceivable side effects (thus unblinding the participants) and the worsening of the condition of the treatment group participants. This observation could incorrectly lead them to the conclusion that they were assigned to the control group. This problem was also discussed by Bang *et al.* [9].

### Method 2: Bang's Blinding Index

As explained in the discussion of the previous section, the blinding index of James *et al.* places a lot of value on those participants who plead ignorance regarding their assignment status. Bang *et al.* see this as a limitation [9]. Specifically, it can be argued that the non-decisive, “don't know” response may not express a true lack of knowledge by the corresponding participants, but rather that it is a conservative response, an answer born out of the participants' desire to appear balanced in their judgement. Thus, Bang *et al.* propose an alternative statistic which instead most heavily weights the contribution of *decisive responses*. In addition, because decisive responses can be in either the positive or the negative direction (i.e. belief in treatment or control intervention), Bang's blinding index is asymmetrical and can be applied separately to treatment and control groups. For a 3-tier auxiliary questionnaire, in its simplest form the index for the treatment group is defined as:
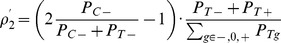
(5)and similarly for the control group:
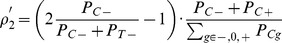
(6)The general form of this index, which applies weighting to different responses in a manner similar to James *et al.* can be found in the original paper [9].

The behaviour of this index can be seen in Figure 1(b) which plots it against the proportions of indecisive responses and correct guesses. It is readily apparent that the plot has a form very different from that in Figure 1(a) showing the corresponding variation of 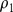. Firstly, note that unlike 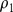, the range of values for 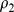 is 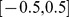. The value of 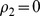 indicates perfect blinding, 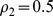 an unblinded trial and 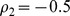 an unblinded trial with incorrect assignment association, as discussed previously.

As the plot shows, this index achieves its perfect blinding value only when 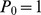. Unlike 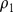, the case when 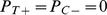 does not necessarily result in perfect blinding. Also, 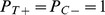 and 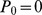 deems the trial unblinded, as does 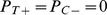 and 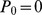 but with the incorrect assignment association. Contrast this with the corresponding value of 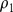.

## Limitations of the Current Best Standards

In the preceding sections we described the two statistics, blinding indexes, most widely used in practice to assess the level of blinding in controlled clinical trials. To highlight and motivate the contribution of the present work, we now analyze both the inherent and practical limitations of the aforesaid methodologies.

### Adjustment of Free Parameters

One of the most obvious difficulties encountered when applying either of the described blinding indexes concerns the need to choose appropriate values for the free parameters in Equation (3), and Equations (5) and (6) in their general form. These are the weighting constants 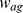. Recall that their purpose is to scale the relative contributions of different responses. For example, James *et al.* propose the ratio of 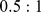 or 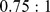 of weights corresponding to, respectively, incorrect assignment guesses and “don't know” responses. Equally, if a questionnaire with more than 3 choices is used, the contribution of the participant's response is scaled according to the corresponding level of conviction expressed. Thus, qualitatively speaking, a response indicating a weak belief in, say, control group assignment, could be interpreted to fall somewhere between a “don't know” response and a strong belief in control group assignment.

Although not without an intuitive appeal, a thorough analysis of this *ad hoc* approach reveals a series of problems, both inherent and practical. Firstly, there is no objective underlying mechanism which would explain why the contributions of different responses should be combined linearly at all. What is more, even if linear combination is adopted, it is inherently the case that there is no principled method of choosing the values of the weighting constants – the lack of observable “ground truth” means that it is not possible to objectively compare the quality of different predictions. The last subtle point but of pervasive practical importance, is that the values of “best” weighting constant ratios are likely to differ from trial to trial, that is, depending on the nature of the administered treatment and the implementation of the control intervention.

All of the aforestated difficulties encountered in the choice of the free parameters necessary to compute the two blinding indexes become even more obvious, practically significant and complex as the questionnaire becomes more detailed (for example by using a 9-tier feedback or by asking the participants to rate the confidence of their response on a scale of 1 to 10, say). Consequently and contrary to what ought to be the case, having better, more detailed feedback data can actually result in a *worse* assessment of blinding due to inappropriate free parameter choices.

### Interpretation of Participants' Feedback

It is important to highlight that both the index of James *et al.* as well as that of Bang *et al.* use the same type of feedback data collected from the trial participants – the participants' stated belief regarding their trial group assignment and the degree of confidence in this belief. Where the two approaches differ in is the *interpretation* of the participants' answers.

James *et al.* interpret the non-decisive, “don't know” response as indicative of true lack of knowledge regarding the nature of the intervention (treatment or control). If the trial participants are ignorant of their group assignment, it is assumed that they have indeed been blinded. Consequently, the index of James *et al.* most heavily relies on the proportion of the non-decisive participants. However, as Bang *et al.* point out, the “don't know” response may not truly represent lack of knowledge. Instead, this response may be seen as a conservative one, reflecting the participants' desire to appear balanced in their judgement or indeed the response that the participants believe would please the trial administration staff the most. Thus, Bang's blinding index mostly relies on the responses of those trial participants who did express belief regarding their group assignment. Blindness is measured by comparing the observed statistics of decisive responses with those expected from an ideal, fully blinded trial. However, this interpretation of participants' responses is readily criticized too. As Hemiliä amongst others notes, because the participants' feedback is usually collected *post hoc* i.e. after the trial, it is possible that even a perfectly blinded subject, who thus did not experience the placebo effect, becomes aware of the correct assignment by virtue of observing the effects (or lack thereof) of the assigned intervention [10]. Considering the same issue, Henneicke-von Zepelin [11] suggested that auxiliary data should be collected before or shortly after the commencement of a trial. However, this is in most cases unsatisfactory as the participants would not have yet been exposed to any unblinded aspects of the trial. As we demonstrate in the next section, the approach proposed in this paper avoids this problem.

### Sensitivity to Small Input Differences

Both James *et al.* and Bang *et al.* establish the level of blindness in a trial by computing a statistic, the blinding index, and then comparing it with a predefined threshold. For example, if 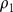 is smaller than the threshold value, the trial is considered insufficiently well blinded; if the blinding index exceeds the threshold, the trial is considered sufficiently well blinded. The value of 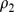 is interpreted in the same way, with the difference that thresholding is done in the opposite direction and on the absolute value of the index.

This hard thresholding whereby a trial is considered either sufficiently well blinded or not means that in some instances the outcome of the blinding assessment can exhibit high sensitivity to small differences in participants' responses. The response of a single individual can change the assessment outcome. Yet, such binarization in some form is necessitated by the nature of the blinding indexes because neither of the two described statistics has a clear practical interpretation in the clinical context.

The task of choosing the value of the aforesaid threshold suffers from much the same problems as the task of selecting the values of the weighting constants, discussed in the “Adjustment of Free Parameters” section – inherently, there is no objective and meaningful way of defining the optimal threshold value, and the value actually selected by the practitioner is likely to vary from trial to trial.

### Inference Atomization

**Figure 2:**
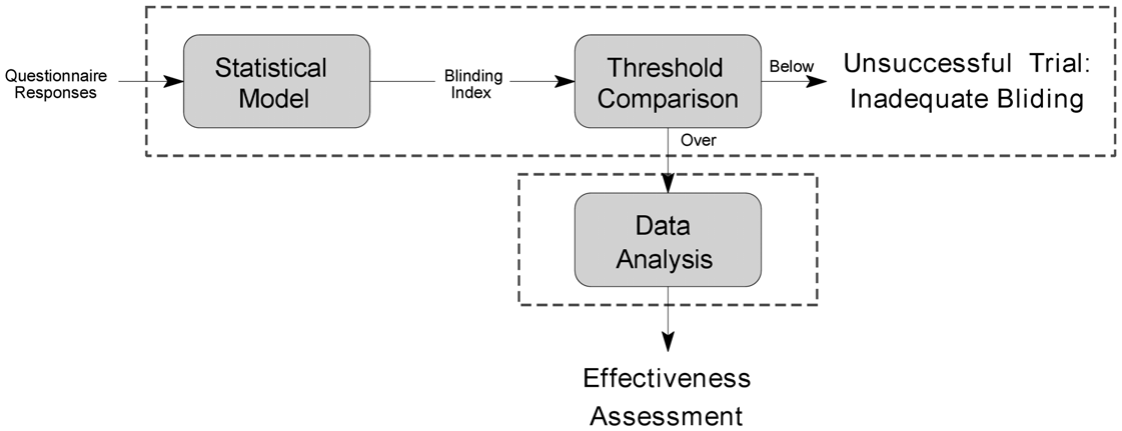
The current clinical practice separates the task of assessing the degree of blinding in a trial from the comparative analysis of the effectiveness of different interventions studied. The effectiveness of the treatment of interest is only analyzed if the trial is first deemed to have been sufficiently blinded. In contrast, the proposed methodology concurrently employs all available information. By doing this, our approach is able to deal with the entire continuum of different levels of blinding, never discarding information.

The problem of high sensitivity to small input differences considered previously is but one of the consequences of the *inference atomization*. To clarify the issue at hand, consider the diagram in Figure 2. This diagram summarizes the process of trial data interpretation, placing the assessment of blinding in the overall clinical context. Specifically, observe that the analysis of the trial outcome data is separated from the blinding assessment. If the blinding index falls short of the predetermined threshold, regardless of by how much, the strength of evidence of data is brought into question and the trial may need to be repeated. On the other hand, if the blinding index exceeds the threshold, the analysis of data is performed in the same manner regardless of the actual value of the index, that is, regardless of whether it is just above the threshold or if it indicates perfect blinding.

The variety of problems that emerges from the atomization of different statistical aspects of a trial is inherently rooted in the very nature of the framework adopted by James *et al.* and Bang James *et al.* alike. As stated earlier, neither of the two indexes has a clear practical interpretation in the clinical context. For example, neither tells the clinician the probability that a particular portion of the participants were unblinded, nor the probability of a particular level of unblinding. Instead, from the point of view of a clinician, the blinding index behaves like a black box which deems the trial well blinded or not, with little additional insight.

## Methods

Having analyzed the limitations of the current clinical practices used to assess blinding, we now turn our attention to the most important contribution of this paper – a principled method for inference from collected trial data. We first introduce a statistical model underlying our approach, followed by the key results. For clarity, the full mathematical derivation of all the results is included in Appendix.

### Study Design and Outcome Model

As we demonstrated in the previous section, many of the problems of the approaches proposed by James *et al.* and Bang *et al.* inherently stem from the underlying statistical model. Although our approach uses the same type of participants' feedback data, our statistical model differs significantly from that employed in previous works.

In the general case, the effectiveness of a particular intervention in a trial participant depends on the inherent effects of the intervention, as well as the participant's expectations (conscious or not). Thus, in the interpretation of trial results, we separately consider each population of participants which share the same combination of the type of intervention and the expressed belief regarding this group assignment. For example, when a 3-tier questionnaire is used in a trial comparing the administration of the treatment of interest and control, we recognize 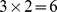 sub-groups:

- participants of the control group who believe they were assigned to the control group (subgroup 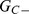),
- participants of the control group who are unsure of their group assignment (subgroup 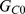),
- participants of the control group who believe they were assigned to the treatment group (subgroup 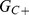),
- participants of the treatment group who believe they were assigned to the control group (subgroup 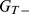),
- participants of the treatment group who are unsure of their group assignment (subgroup 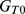), and
- participants of the treatment group who believe they were assigned to the treatment group (subgroup 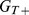).

**Figure 3:**
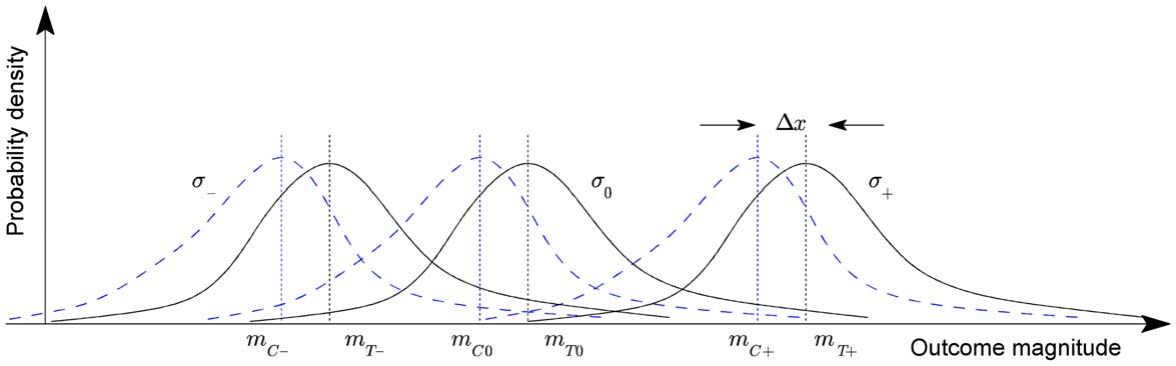
A conceptual illustration of the proposed statistical model for the 3-tier participants' feedback questionnaire: (i) belief in treatment assignment, (ii) belief in control assignment and (iii) uncertain (“don't know”). Dotted and solid lines show respectively the probability density functions of the measured trial outcome across individuals in the three control and treatment sub-groups. Parameters 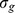 and 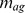 denote the corresponding standard deviation and the mean of each sub-group, and 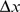 the differential effect of treatment.

This is conceptually illustrated in Figure 3. In the general case, for an 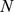-tier questionnaire and 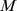 different intervention types, we can distinguish between 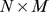 distinct sub-groups of participants.

A key idea of the proposed method is that because the outcome of an intervention depends on both the inherent effects of the intervention and the participants' expectations, the effectiveness should be inferred in a like-for-like fashion. In other words, the response observed in, say, the sub-group of participants assigned to the control group whose feedback professes belief in the control group assignment should be compared with the response of only the sub-group of the treatment group who equally professed belief in the control group assignment. Similarly, the “don't know” sub-groups should be compared only with each other, as should the subgroups corresponding to the belief in the treatment assignment. Ideas similar in spirit were expressed by Berger in the consideration of the related problem of so-called selection bias and specifically the Berger-Exner test [12]. However, the manner in which these ideas are formalized statistically in the present paper is entirely different, the methods described by Berger sharing many of the weaknesses of the approaches of James *et al.* and Bang *et al.*, which were analyzed in detail in the “Limitations of the Current Best Standards” section. The proposed approach is formalized next.

### Inference

Consider two corresponding sub-groups, that is, sub-groups corresponding to different types of received intervention but the same response in the participants' feedback questionnaire. Furthermore, let the benefit of an intervention observed in a particular participant be expressed as a real number 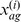. Thus, and without loss of generality, a greater 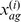 indicates greater benefit. For example, 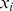 may represent the amount of fat loss in a fat loss trial, the reduction in blood plasma LDL in a statin trial etc. Our goal is to infer 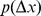, that is, the probability density function over the difference 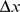 in the benefit observed across the two compared sub-groups.

Let 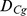 be the trial outcome data collected from a control sub-group and 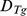 of the matching treatment sub-group.
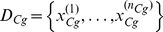
(7)

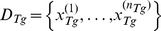
(8)Then, if 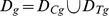 is the totality of all data of participants who believe they were assigned to the group 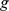, by applying Bayes rule we can write the following:
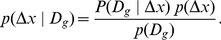
(9)Modelling the response of each sub-group using a normal (i.e. Gaussian) distribution
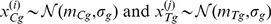
(10)and remembering that for the underlying distributions it holds that 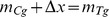, allows us to further write
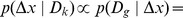
(11)

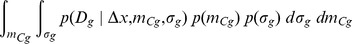
(12)where 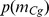 is a prior on the mean of the control sub-group and 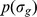 a prior on the standard deviation within sub-groups. What Equation (12) expresses is the process of probability density function marginalization over nuisance variables 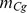 and 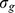. Since the values of these latent model variables are unknown, marginalization takes into account all of the possibilities and weights them in proportion to the supporting evidence.

As shown in full detail in Appendix 0, when two corresponding sub-groups of participants are considered, for uninformed priors over 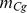 and 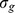, the posterior distribution of 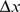 is given by:
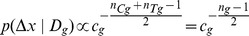
(13)where constant scaling factors have been omitted for clarity, and
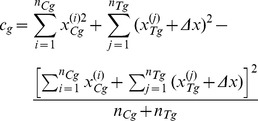
(14)Extending to the joint inference over the entire data corpus, the posterior can be computed simply as a product of all sub-group pair posteriors (up to a scaling constant):
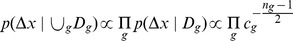
(15)The estimate of the posterior distribution of 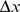 in Equation (15) is the best estimate that can be made using the available data, and it is of the most interest to the clinician. However, as we will discuss in the “Discussion” section, both Equation (13) and Equation (15) have significance in the interpretation of trial results and their joint consideration can be used to reveal important additional information about the effectiveness of the treatment.

## Results

Certain advantages of the proposed methodology, in comparison to the approaches of James *et al.* and Bang *et al.* are *ipso facto* inherent in the theory developed in the preceding sections. The absence of free parameters is one such advantage. Other claimed properties of the method, such as its robustness to small input differences (i.e. differences in the patients' feedback responses), are not immediately obvious. Thus, in this section we present the results of a series of experiments which demonstrate the superiority of the proposed method.

### Evaluation Methodology

In contrast to the methods of James *et al.* and Bang *et al.* which do not attempt to infer any objective and measurable quantity, the proposed approach pools all available data (trial outcomes and auxiliary questionnaire feedback) in an effort to evaluate robustly the effectiveness of the studied treatment. This feature of our method allows us to directly evaluate its performance. Specifically, we employ a computer-based simulation whereby data is first randomly (or rather pseudo-randomly) generated using a statistical model with adjustable parameters, followed by the application of the proposed method which is used to infer the said parameters. The values inferred by our method can then be directly compared with their known true values.

#### Experiment 1: Reference

For our first experiment, we simulated a trial involving 200 individuals, half of which were assigned to the control and half to the treatment group. For each of the groups, 60% of the participants were taken to be in the “undecided” subgroups 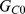 and 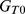. The remaining 40% of the participants was split between correct and incorrect guesses of the assigned intervention in proportion 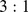. This is summarized in Table 2. In this initial experiment we assume that all participants correctly disclosed their belief regarding which group they were assigned to. Note that this assumption is done purely in the process of generating data for the experiment – neither this nor any of the preceding information is used by our method to analyze the outcome of the trial.

**Table 2:** A summary of the trial size, the split of participants into groups and their auxiliary subgroups, used to generate data for the initial experiment.

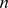	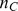	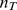	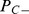	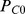	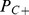	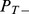	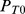	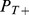
200	100	100	0.3	0.6	0.1	0.1	0.6	0.3

We set the differential effect of treatment to 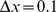 and the standard deviation of variability within each of the assignment-response subgroups (please refer back to the “Study Design and Outcome Model” section) also to 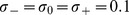. Relative to genuine lack of belief in either control or treatment group assignments, belief in control group assignment was set to exhibit negative effect of magnitude 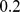 (i.e. 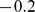) and that in treatment group assignment a positive effect of magnitude 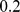. Experimental parameters are summarized in Table 3.

**Table 3:** The ground truth parameters of the underlying distributions used to generate intervention outcomes for participants in our simulated trial.

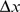	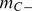	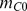	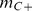	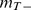	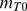	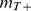	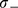	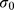	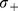
0.1	−0.2	0.0	0.2	−0.1	0.1	0.3	0.1	0.1	0.1

The reader may find it useful to refer back to Figure 3.

**Figure 4:**
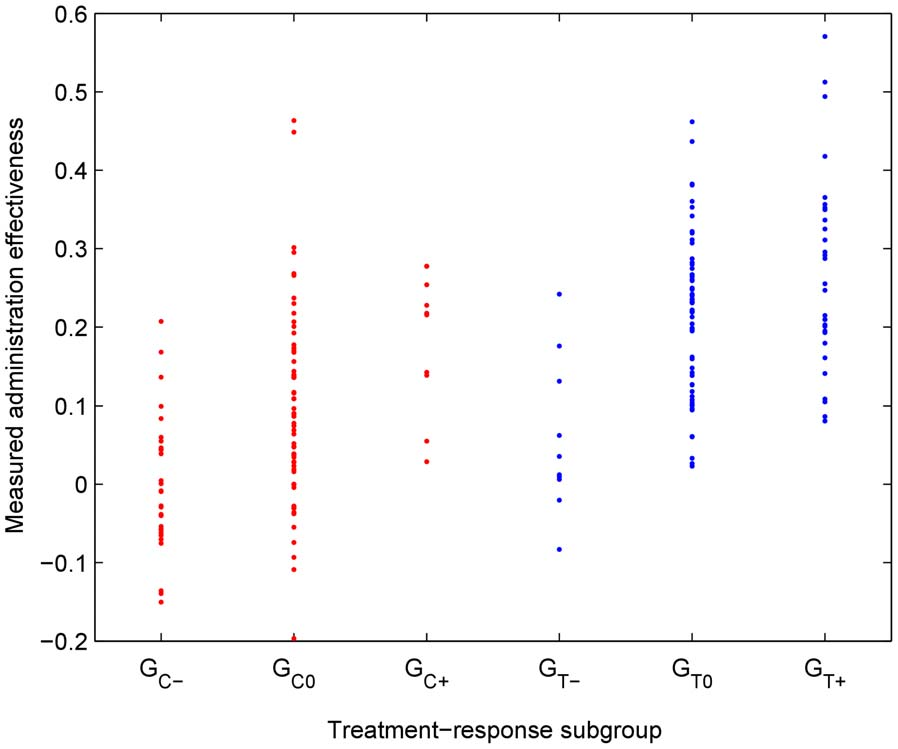
The spread of observed (measured) treatment responses (vertical axis) of the participants in the simulated reference trial across the six sub-groups (horizontal axis; see the “Study Design and Outcome Model” section).

Intervention outcomes were then generated by repeated random draws from the corresponding distributions. For example, the outcome associated with a participant in the group 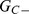 was determined by a random draw from the normal distribution 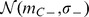. The outcomes thus obtained are detailed in Table 4 and visually illustrated in Figure 4.

**Table 4:** Automatically generated data for the simulation trial used to assess the proposed method.

Group	Subgr.	Observed intervention outcome/effectiveness (per individual)									
Control group	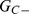	0.001	0.010	−0.029	−0.062	−0.150	−0.058	0.207	−0.065	0.046	−0.075
		−0.040	−0.009	0.060	−0.038	−0.139	0.084	−0.070	0.055	0.168	0.005
		0.044	−0.040	−0.054	−0.136	−0.008	−0.027	0.0465	−0.057	0.136	0.039
	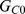	0.268	0.173	−0.109	0.201	0.135	0.230	0.096	0.064	−0.031	0.116
		0.000	0.034	−0.030	−0.036	0.023	0.090	0.038	0.170	0.218	0.048
		0.207	0.028	−0.027	−0.000	0.090	0.237	0.087	0.048	0.109	0.139
		0.156	−0.038	0.266	0.117	0.177	0.074	0.463	−0.004	0.138	−0.093
		0.144	−0.197	0.295	−0.055	0.069	0.039	0.135	0.019	0.016	0.449
		0.301	0.192	0.109	0.037	0.168	0.136	0.052	0.029	−0.074	0.078
	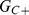	0.218	0.277	0.138	0.142	0.029	0.254	0.278	0.0549	0.228	0.215
Treatment group	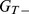	0.010	0.131	0.006	−0.020	0.062	0.036	0.176	0.012	0.242	−0.083
	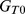	0.026	0.221	0.248	0.033	0.240	0.148	0.108	0.287	0.162	0.095
		0.097	0.437	0.061	0.462	0.250	0.102	0.267	0.282	0.100	0.264
		0.199	0.241	0.219	0.138	0.023	0.236	0.213	0.112	0.195	0.159
		0.127	0.307	0.204	0.231	0.232	0.360	0.232	0.342	0.382	0.197
		0.353	0.261	0.222	0.104	0.381	0.280	0.232	0.322	0.267	0.118
		0.259	0.234	0.249	0.242	0.142	0.311	0.320	0.060	0.219	0.275
	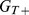	0.336	0.296	0.291	0.209	0.081	0.086	0.311	0.203	0.325	0.193
		0.418	0.287	0.195	0.161	0.255	0.215	0.356	0.352	0.209	0.714
		0.109	0.247	0.141	0.571	0.105	0.179	0.201	0.350	0.365	0.512

**Figure 5:**
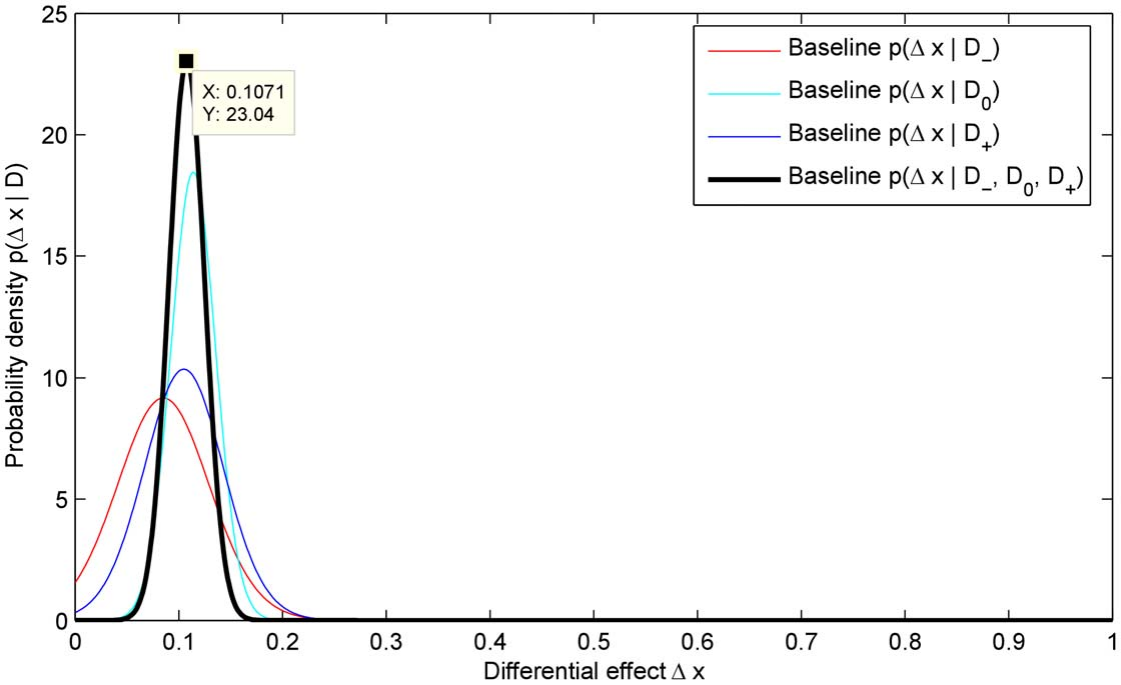
The three sub-group posteriors, 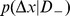, 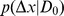 and 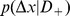, and the joint posterior 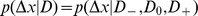. Also marked is the mode of the posterior 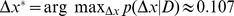.

The result of applying the proposed method is summarized in Figure 5 which plots the posteriors corresponding to the three subgroups matched by the patients' belief and the amalgamated posterior. The same plot also marks the *maximum a posteriori* (MAP) value 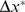 of the estimate of the differential effectiveness of the treatment:
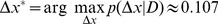
(16)which is very close to the true value of 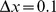.

In comparison, when the differential effectiveness is estimated by subtracting the mean response of the control group from that of the treatment group, without the use of our matching sub-groups based statistical model, the estimate 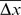 is:
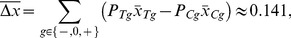
(17)where 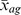 is the mean effectiveness across the subgroup 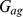:
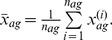
(18)Finally, the corresponding values of the blinding indexes proposed by James *et al.* and Bang *et al.* are:
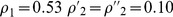
(19)Notice that the former indicates a level of blinding roughly half way between a perfectly blinded and unblinded trial, while the latter deems the trial nearly perfectly blinded.

#### Experiment 2: Conservative Distortion

In the “Interpretation of Participants' Feedback” it was explained why the participants in a trial may not be fully honest in their auxiliary questionnaire feedback and how this presents a major problem in formulating a universally correct statistical model. For example, there may be a conservative drift towards the middle whereby participants falsely declare uncertainty towards their group assignment out of desire of appearing balanced in their judgement.

It was argued on theoretical grounds that the proposed methodology presents a universal framework for controlled trial assessment and analysis, and as such is robust to the aforementioned phenomenon. Here we investigate this claim experimentally.

We modify the baseline experiment described in “Experiment 1: Reference” by simulating conservative behavioural tendency of participants in a trial. This was achieved by randomly choosing individuals from decisive subgroups (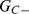, 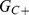, 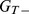 and 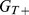) and re-assigning them to their corresponding indecisive subgroup *without* changing their treatment's observed effectiveness. Thus, a randomly chosen participant from subgroups 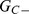 or 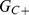 would be assigned to the subgroup 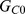 and one from subgroups 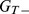 or 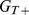 to 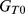. The probability of re-assignment was set to 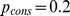.

**Figure 6:**
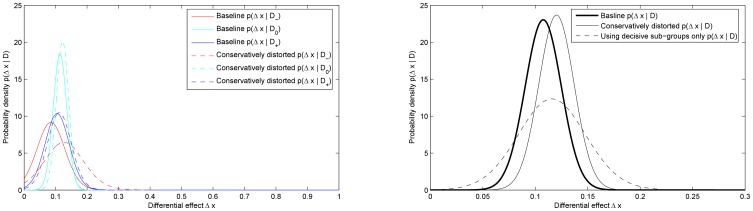
Experiment 2. (a) Posteriors for the differential effect treatment 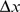 computed using the data 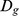 of each experimental sub-group comprising control and treatment individuals matched by their feedback. (b) Posterior for the differential effect treatment 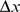 computed using all available data 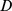 (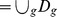).

As before, we applied the proposed method on the modified data and display the key results in Figure 6. In addition to the new subgroup posteriors (dotted lines), for comparison in Figure 6 (a) we also show the three initial subgroup posteriors from Experiment 1 (solid lines). The baseline (thick solid line) and new (thin solid line) amalgamated posteriors are shown in Figure 6 (b). Figure 6 (b) also shows the semi-amalgamated posterior obtained using only decisive subgroups which, by experimental design, comprise data of only those individuals which honestly disclosed their belief of group assignment. The new MAP value for the differential effectiveness using the amalgamated posterior can be seen to be 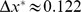 and that using the semi-amalgamated posterior 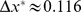. In the “Further Clinical Insight” section we will show how the difference in statistical features of sub-group posteriors can be used to select the most reliable posteriors to amalgamate, as well as to reveal additional insight into the nature of the studied treatment and the blinding in the trial.

The conventionally measured mean differential effect remains unaffected by the conservative distortion; however, the assessment of sufficient blinding does not (this is discussed in detail in the “Discussion” section).

#### Experiment 3: Asymmetric Progressive Unblinding

One of the unappealing consequences of inference atomization, i.e. the separation of blinding assessment and the actual trial outcome, adopted by James *et al.* and Bang *et al.* alike, becomes readily apparent when the effects of small differences in the patients' feedback responses are considered. On the one hand, as long as the blinding index stays above or below the chosen threshold, a small difference in the patients' feedback (e.g. the change in a single individual's response) is of no consequence to the subsequent analysis of the actual trial data. On the other hand, for the values of the blinding index near the chosen threshold, an equally small input difference can result in the complete rejection of trial data, due to insufficient blinding. In this experiment and in Experiment 4 we examine the behaviour of the proposed method as its input in the form of the trial participants' auxiliary data is progressively altered.

Starting with the baseline setup of Experiment 1, we simulate unblinding of previously undecided individuals of the treatment group. In other words, in each turn we re-assign an individual from the subgroup 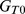 to the subgroup 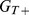 and compute the novel distribution for 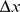. We call this experiment *asymmetric progressive unblinding* because only the treatment group participants are being unblinded (symmetric unblinding is considered in “Experiment 4: Symmetric Progressive Unblinding”).

**Figure 7:**
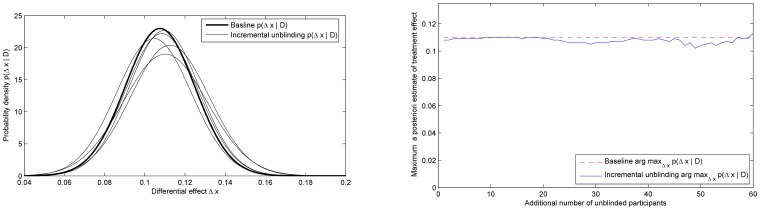
Experiment 3. (a) Initial posterior (bold line) and the sequence of posteriors as “don't know” responders in the treatment group are progressively unblinded. Each next posterior plot corresponds to additional 10 “don't know” respondents who become members of the sub-group which correctly identified their group membership. (b) The *maximum a posteriori* estimate of the treatment effectiveness as the participants assigned to the treatment group are progressively unblinded. Observe the robustness of the proposed approach, witnessed by little effect that unblinding has on the estimate (standard deviation of the *maximum a posteriori* estimate: 0.0054); compare with Figure 8.

**Figure 8:**
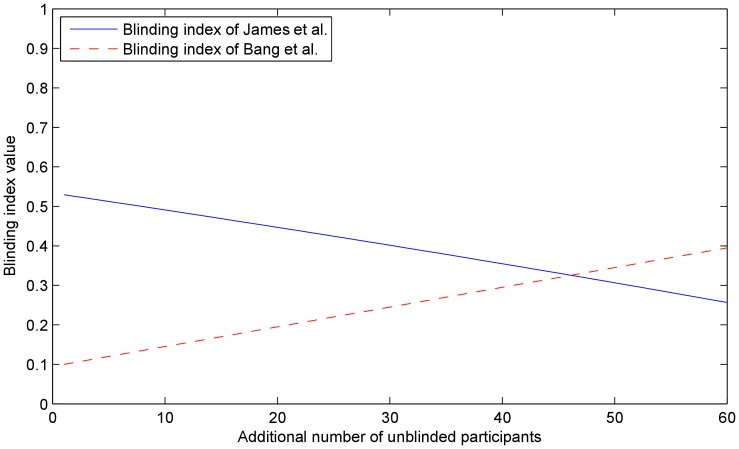
Experiment 3. The values of the blinding indexes proposed by James *et al.* (blue line) and Bang *et al.* **(**red line), computed at each step of the progressive unblinding of the participants assigned to the treatment group. As expected, both indexes dramatically change in value and in this case agree that the level of blinding in the trial has decreased. Compare this with the robustness of the proposed approach in Figure 7.

Figure 7 (a) shows the initial posterior 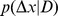 (bold line) and a series of posteriors after a greater and greater number of previously blinded participants were unblinded. The obtained posteriors demonstrate the robustness of the proposed approach, with small random variations as expected in any practical experiment involving stochastic data. The resilience of the method is further corroborated in Figure 7 (b), which shows the *maximum a posteriori* estimate of the effectiveness of the treatment (i.e. the mode of the posterior). This estimate also only shows small random perturbations, with the corresponding standard deviation of only 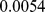. The plots in Figure 8 show the variation of the two blinding indexes throughout the experiment. As expected from the change in the participants' auxiliary data, both indexes change in value dramatically. The index of James *et al.* decreases, while that of Bang *et al.* increases in absolute value, indicating agreement on the lowered level of blinding.

#### Experiment 4: Symmetric Progressive Unblinding

Finally, in the last simulated experiment, we consider the effects of unblinding previously blinded trial participants from *both* the treatment and the control group. Notice that this results in a progressively more unbalanced distribution of individuals in the two groups across the corresponding sub-groups. This is important because of the matching sub-group comparison at the heart of the proposed approach (see the “Methods” section) and is discussed in detail in the next section.

As in Experiment 3 we start with the baseline setup of Experiment 1 and simulate unblinding of previously undecided individuals of the treatment group. In each turn of progressive unblinding we re-assign an individual from the subgroup 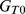 to the subgroup 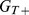 and an individual from the subgroup 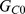 to the subgroup 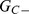, and compute the novel distribution for 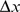. We call this experiment *symmetric progressive unblinding* because both the treatment group and the control group participants are being unblinded (asymmetric unblinding was considered in “Experiment 3: Asymmetric Progressive Unblinding”).

**Figure 9:**
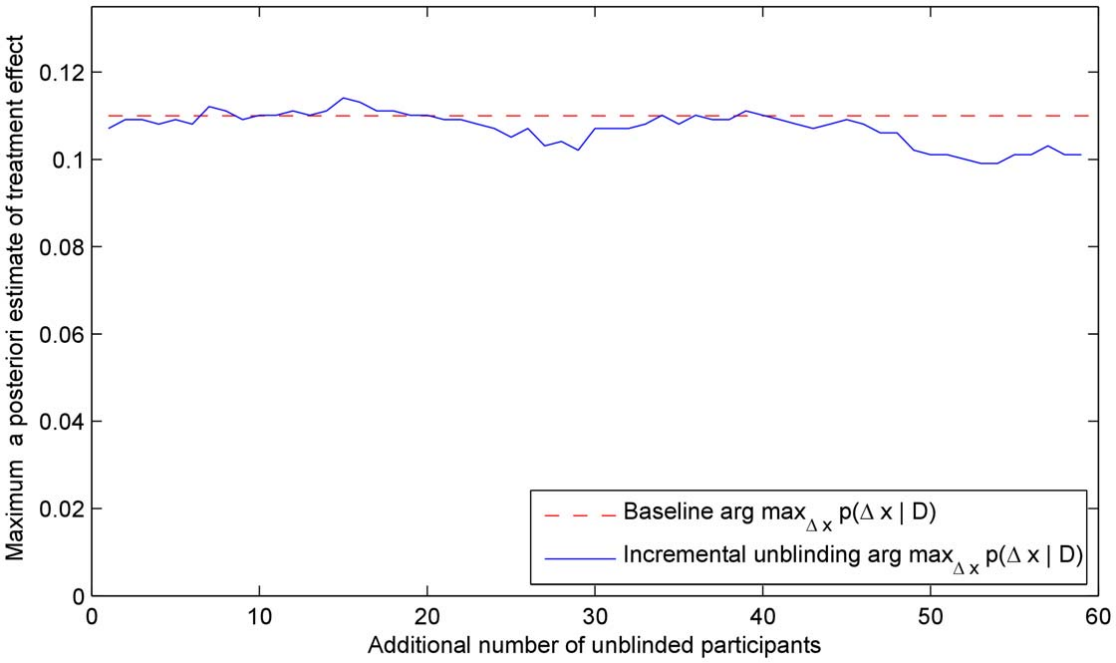
Experiment 4. The *maximum a posteriori* estimate of the treatment effectiveness as the participants assigned to both the treatment and the control groups are progressively unblinded (compare with the previous, asymmetric blinding experiment and Figure 7). As before, the proposed method exhibits remarkable robustness.

**Figure 10:**
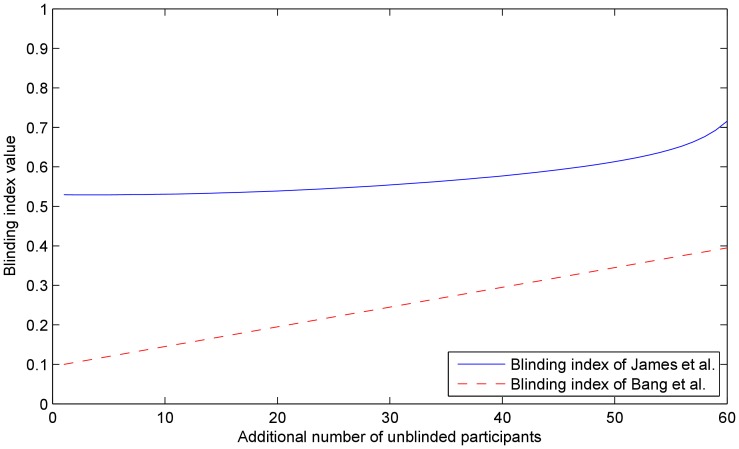
Experiment 4. The values of the blinding indexes proposed by James *et al.* (blue line) and Bang *et al.* (red line), computed at each step of the progressive unblinding of the participants assigned to the treatment group. While both indexes again dramatically change in value, interestingly they also show disagreement on the direction of change in blinding. This is a consequence of the difference in the underlying assumptions used to interpret the participants' auxiliary data. Compare this with the robustness of the proposed approach in Figure 9.

We illustrate the robustness of the method by plotting the *maximum a posteriori* estimate of the effectiveness of the treatment (i.e. the mode of the posterior) in Figure 9. As before, the estimate only shows small random perturbations, as expected in any experiment with a stochastic nature and is to be contrasted with the plots in Figure 10 which show the changes in the two blinding indexes throughout the experiment. Again, with the change in the participants' auxiliary data, both indexes also change in value. It is insightful to observe that unlike in Experiment 3, in this instance the values of the two indexes do not exhibit agreement on the direction of change of the level of blinding. This reflects the importance that the auxiliary data interpretation plays in the methods of both James *et al.* and Bang *et al.*

## Discussion

In the “Limitations of the Current Best Standards” section we identified and discussed the key limitations of the currently employed practices for assessing and accounting for imperfect blinding in controlled clinical trials. Two key problem areas were identified. The first of these is the separation of blinding assessment using auxiliary trial data and the analysis of the main outcome of the trial. The other major limitation of the current practices is rooted in the interpretation of auxiliary data and specifically the seemingly unavoidable need to model patients' behaviour.

The separation of blinding assessment and main outcome analysis, or inference atomization as we term it here, gives rise to a number of undesirable consequences. For example, the assessment of blinding of a trial can flip from successful to failed (or *vice versa*) as a result of alteration in the feedback response of only a single participant. Such sensitivity to small input differences means that even an elementary change in the participants' feedback may result in all of the main trial data collected being thrown away. This is clearly illustrated in Experiments 3 and 4 which demonstrate the change in value of both blinding indexes as the trial is gradually unblinded.

On the other hand, as long as the trial is deemed successful, no information about the extent of blinding is propagated and accounted for in the subsequent phase when the main trial data is analyzed. For example, despite the change in feedback responses between Experiment 1 and Experiment 2, and with it the change in the values of blinding indexes, the entirety of the main trial data in the two experiments was left unaltered. Since the assessment of blinding is statistical in nature, it is not possible to identify participants which may not have been blinded well enough. Thus, data from a perfectly blinded trial is interpreted and processed in the same way as of a barely sufficiently blinded trial (i.e. one with a blinding index just exceeding a set threshold). This is in sharp contrast with the proposed method. Because feedback responses and the main trial data are analyzed in unison, the output of the proposed method is unaffected by the unblinding in both Experiment 3 and Experiment 4 (save for stochastic perturbations).

### Degenerate Cases

One of the key ideas behind the present method is that it is meaningful to compare only the corresponding treatment and control participant sub-groups, that is, sub-groups matched by their auxiliary responses. As noted in the “Limitations of the Current Best Standards” section, while a greater number of subgroups may provide more precise auxilliary/blinding information, the introduced partitioning of data decreases the statistical strength of each comparison of the corresponding sub-groups which results in a posterior with a wider spread. In an extreme case, a particular sub-group may be empty. In other words, it is possible that none of the participants of the treatment or the control group expressed a particular belief regarding their treatment assignment. Although this may appear as a problem at first, a more careful examination of such cases reveals that this is not so.

Firstly, note that whenever at least one pair of matching sub-groups is non-empty, the proposed method is able to compute a meaningful estimate of differential treatment effectiveness. In instances when there are no non-empty matching sub-groups, the nature of degeneracy can provide useful insight to the clinician.

The absence of individuals in the 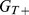 sub-group may indicate that the participants assigned to the treatment group have either been poorly blinded but misidentified the received treatment, or that the treatment was vastly ineffective and was recognized as such by the participants assigned to it. Similarly, the absence of individuals in the 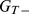 sub-group may indicate that the participants assigned to the treatment group have either been poorly blinded and correctly identified the received treatment, or that the treatment was obviously effective. In all cases, because degenerate data is trivial to recognize, the clinician is immediately made aware of the presence of a major flaw in the experimental design. The clinician can then identify the cause of degeneration using own knowledge of the administered interventions, and the statistics of both auxiliary responses and trial outcomes.

### Further Clinical Insight

In the “Inference” section we derived posteriors corresponding both to only a single pair of corresponding sub-groups in Equation (13) and to the entirety of data, that is, all sub-groups in Equation (15). While the latter of these is of primary interest, the clinician can derive further useful insight into the nature of studied treatment by comparative examination of sub-group posteriors too.

The least interesting case is when the sub-group posteriors and the total posterior exhibit similar characteristics (e.g. the location of the mode). However, consider the case when that is not so. For example, let us say that the posterior corresponding to the two matching “don't know” subgroups has the mode near 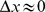 and the total posterior has a decidedly positive mode (with suitably small standard deviations, to make the observation statistically significant). This could indicate that there may be so-called “non-responders” in the treatment group, i.e. individuals which did not respond positively to the treatment which in most people does produce a positive result [13], [14]. Similar arguments can be made by considering differences between other sub-group posteriors. Ultimately, the exact interpretation is in the hands of the clinicians who should use their insight into the nature of the administered interventions to infer further information of this type.

## Summary and Conclusions

This paper examined the problem of assessing the extent of blindness in a clinical trial. Currently, this is achieved by collecting auxiliary data in the form of a questionnaire which asks the trial participants which group (treatment or control) they think they were assigned to. Methods in use today employ this data to compute a statistic, a blinding index, which is then compared against a threshold leading to a crisp positive or negative decision on whether the trial was sufficiently blinded.

Our first major contribution was to demonstrate a series of flaws in blinding index based approaches. The main flaws are: (i) the presence of *ad hoc* free parameters in the statistical model used to derive a blinding index, (ii) the non-universality of the assumptions used to interpret the auxiliary questionnaire responses, (iii) the sensitivity of blinding assessment to small changes in participants' questionnaire responses, and (iv) the sequential separation of inference concerning the extent of trial blindness and the assessment of differential effectiveness of the treatment. Thus, a novel framework was proposed.

We too adopt an auxiliary questionnaire of the same form as currently in use but describe a statistical model which does not rely on the assumptions made in previous work that lead to the aforementioned difficulties. At the centre of the idea is that the comparison of the treatment and control groups should be done in like-for-like fashion, giving rise to the partitioning of participants into sub-groups, each sub-group sharing the same intervention and auxiliary responses. A Bayesian framework was used to interpret jointly the auxiliary and trial outcome data, giving the clinician a meaningful and readily understandable end result. The effectiveness of our method was demonstrated empirically in a simulation study, which showed its robustness in a variety of scenarios. In addition, it was shown how our method can be used to reveal additional important information regarding the effectiveness of the trial, such as groups of non-responders.

Lastly, our work strongly highlights the need for open reporting of trial results. Specifically, although the proposed method uses the same type of auxiliary data as the previously proposed methods, it is necessary that the auxiliary and trial outcome responses are matched by individual participant, rather than aggregated. At present, this detail is not widely reported nor readily made available even upon request.

## Derivation of the Key Equations

Consider two matching sub-groups 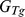 and 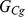, one corresponding to the treatment group and one to the control group, such as 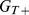 and 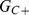. Our goal is to infer the posterior distribution 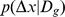 for the differential effect of treatment 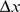 (the difference in the effectiveness between the treatment and the control group) given the main trial data 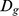 (the observed effectiveness of target and control interventions in the two subgroups). The entire data corpus 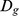 consists of responses of the control subgroup 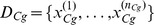 and the responses of the corresponding treatment subgroup 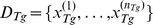 where 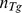 is the number of participants in 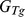 and 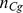 the number of participants in 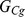. Using Bayes rule, the posterior can be expressed in terms of likelihood 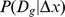 for 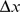 and the priors 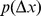 and 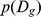:
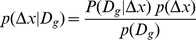
(20)Assuming an uninformed prior for 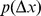 and observing that in this context 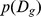 is merely a weighting constant, Equation (20) can be simplified to:
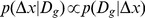
(21)As usual, we assume that the effectiveness response of each trial subgroup obeys a normal distribution. Let the mean outcome for the control subgroup be 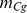 and consequently the mean of the corresponding treatment subgroup 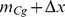, and the standard deviation of outcomes within the sub-groups 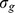. Since we again have no universal reason to expect one value of 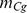 or 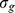 over another, that is, no means of formulating an informed prior on 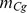 and 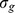, we use the appropriate uninformed priors which are in this case [15]:
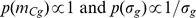
(22)Note that these are improper priors (i.e. they do not integrate to unity). Using a Bayesian approach whereby the unknown latent variables of the model are integrated out:
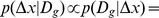
(23)

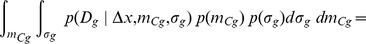
(24)

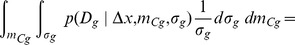
(25)

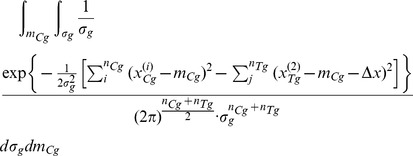
(26)Now consider the term in the brackets of the numerator's exponent in Equation (26). This term can be written as a sum of a square and a constant:
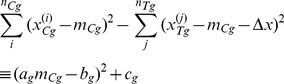
(27)Expanding the left-hand size and equating the coefficients corresponding to different powers of 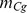 gives the following values for the unknown variables 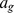, 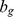 and 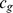 on the right-hand size of the equality:
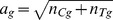
(28)

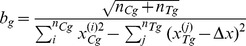
(29)

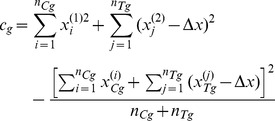
(30)Continuing to use constants 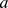, 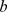 and 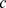 for clarity, Equation (26) can be written as:
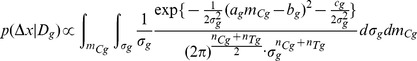
(31)Re-arranging the right-hand size further and integrating out the Gaussian gives:
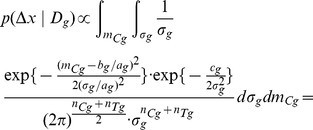
(32)

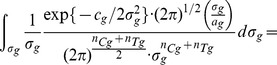
(33)

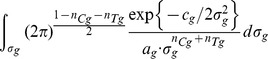
(34)Note that the expression in Equation (34) has the functional form of the inverse gamma distribution:
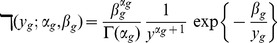
(35)where
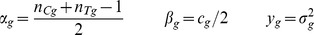
(36)Thus, re-writing Equation (35) and remembering that the inverse gamma distribution must integrate to unity (being a probability density function), gives:
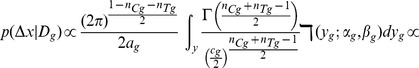
(37)

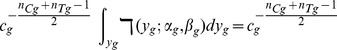
(38)which is the exactly the expression in Equation 13 introduced in the main text in the “Inference” section. QED.
